# Aspects and prospects of preclinical theranostic radiopharmaceutical development

**DOI:** 10.7150/thno.100339

**Published:** 2024-10-07

**Authors:** Bryce J.B. Nelson, Viktoria Krol, Aditya Bansal, Jan D. Andersson, Frank Wuest, Mukesh K. Pandey

**Affiliations:** 1 Department of Radiology, Mayo Clinic, Rochester, MN 55905, USA.; 2 Department of Oncology, Cross Cancer Institute, University of Alberta, Edmonton, Alberta, T6G 1Z2 Canada.; 3 Edmonton Radiopharmaceutical Center, Alberta Health Services, Edmonton, Alberta, T6G 1Z2, Canada.; 4 Cancer Research Institute of Northern Alberta, University of Alberta, Edmonton, Alberta, T6G 2E1, Canada.; 5 Mayo Clinic Comprehensive Cancer Center, Rochester, MN 55905, USA.

**Keywords:** Theranostics, PET, SPECT, Alpha therapy, Beta therapy, Nuclear Medicine

## Abstract

This article provides an overview of preclinical theranostic radiopharmaceutical development, highlighting aspects of the preclinical development stages that can lead towards a clinical trial. The key stages of theranostic radiopharmaceutical development are outlined, including target selection, tracer development, radiopharmaceutical synthesis, automation and quality control, *in vitro* radiopharmaceutical analysis, selecting a suitable *in vivo* model, preclinical imaging and pharmacokinetic analysis, preclinical therapeutic analysis, dosimetry, toxicity, and preparing for clinical translation. Each stage is described and augmented with examples from the literature. Finally, an outlook on the prospects for the radiopharmaceutical theranostics field is provided.

## 1. Introduction

Nuclear medicine involves using unsealed radionuclide sources to diagnose and treat disease 1. An early nuclear medicine development involved the use of ^131^I for treating benign and malignant thyroid disorders, with the first patient treated in 1941 for hyperthyroidism. Since then, ^131^I has been widely used to treat patients with thyroid disorders and remains standard of care today 2. These radionuclides can either be injected as a simple formulation such as [^223^Ra]RaCl_2_ (Xofigo^®^) for palliative treatment of prostate cancer bone metastases 3, or radiolabeled onto targeting vectors to create radiopharmaceuticals, such as [^68^Ga]Ga-PSMA-11 for diagnosing prostate-specific membrane antigen (PSMA) expressing prostate cancer 4.

Technetium-99m (^99m^Tc) became the first workhorse radionuclide for molecular imaging, with a variety of ^99m^Tc radiopharmaceuticals providing functional 3D information to clinicians through single-photon emission computed tomography (SPECT) imaging 5. More recently, positron emission tomography (PET) imaging has improved sensitivity and resolution relative to SPECT imaging 5. PET and SPECT radiotracers have been developed to target specific markers on cancer cells that are upregulated relative to healthy tissue; notably PSMA, which is often overexpressed in prostate cancer 6,7, the somatostatin receptor 2 (SSTR2) which is commonly overexpressed on neuroendocrine tumors 8, and the fibroblast activation protein (FAP) 9, which is overexpressed in various tumor microenvironments.

If a diagnostic radiotracer exhibits a promising biodistribution in SPECT or PET imaging, accumulating primarily at diseased sites with minimal uptake in healthy tissues, it may be a potential candidate for subsequent targeted radionuclide therapy (TRT). When radiotracers are first labeled with a diagnostic PET or SPECT radionuclide to image disease, and the same tracer is subsequently labeled with a therapeutic radionuclide for TRT, this is termed radiotheranostics 10. This precision medicine technique enables non-invasive visualization of malignancies and development of a treatment plan. Ideally, the imaging and therapeutic radionuclides possess a comparable half-life and similar or identical chemistries, since this ensures that the initial diagnostic scan is a close match to the biodistribution of the therapeutic radiopharmaceutical 11. Even though this arrangement is ideal, it is not necessary, as seen with applications pairing together imaging and therapeutic PSMA targeting vectors with significantly different structures and radionuclides. Alternatively, PET or SPECT molecular imaging can be used to track non-radioactive therapeutic vectors, such as cell or antibody-based therapies, in a “hybrid” or “integrated” theranostic approach 12.

Recently, targeted beta minus (β^-^) particle therapy using radiometals such as lutetium-177 (^177^Lu) 13,14 or copper-67 (^67^Cu) 15, and targeted alpha (α) particle therapy using radiometals such as actinium-225 (^225^Ac) 6,16-18 or lead-212 (^212^Pb) 19-22 have shown promise in treating patients with metastatic prostate cancer and neuroendocrine tumors. Diagnostic scans are typically performed with standard PET radionuclides such as ^68^Ga or ^18^F, which have shorter half-lives and different chemistries relative to their therapeutic counterparts 23, or they may be performed with radionuclide imaging surrogates that possess similar half-lives and chemistries to their therapeutic counterparts. Alternatively, radiotracers designed for dual-labeling of diagnostic and therapeutic radionuclides can enable near-identical imaging and therapy biodistributions with chemically dissimilar radionuclides 24,25.

This article aims to provide an overview of theranostic radiopharmaceutical development. Theranostic target selection will be explored, followed by tracer development, radionuclide selection, radiopharmaceutical synthesis, and associated quality control techniques. Then, preclinical radiopharmaceutical analysis will be outlined, including *in vitro* analysis, *in vivo* model selection, preclinical imaging and pharmacokinetic analysis, therapy studies, and dosimetry. Finally, aspects of potential translation to clinical trials will be discussed, with an outlook for the field of radiopharmaceutical theranostics.

## 2. Theranostic target selection

The first step in designing a theranostic radiopharmaceutical involves target selection. Ideally, targets will specifically address an unmet clinical need. Metastatic cancers are one disease area where theranostics holds significant potential, owing to refractory disseminated disease that can render standard-of-care treatments such as surgery, chemotherapy, and external beam radiation therapy less effective 26.

**Figure 1:**
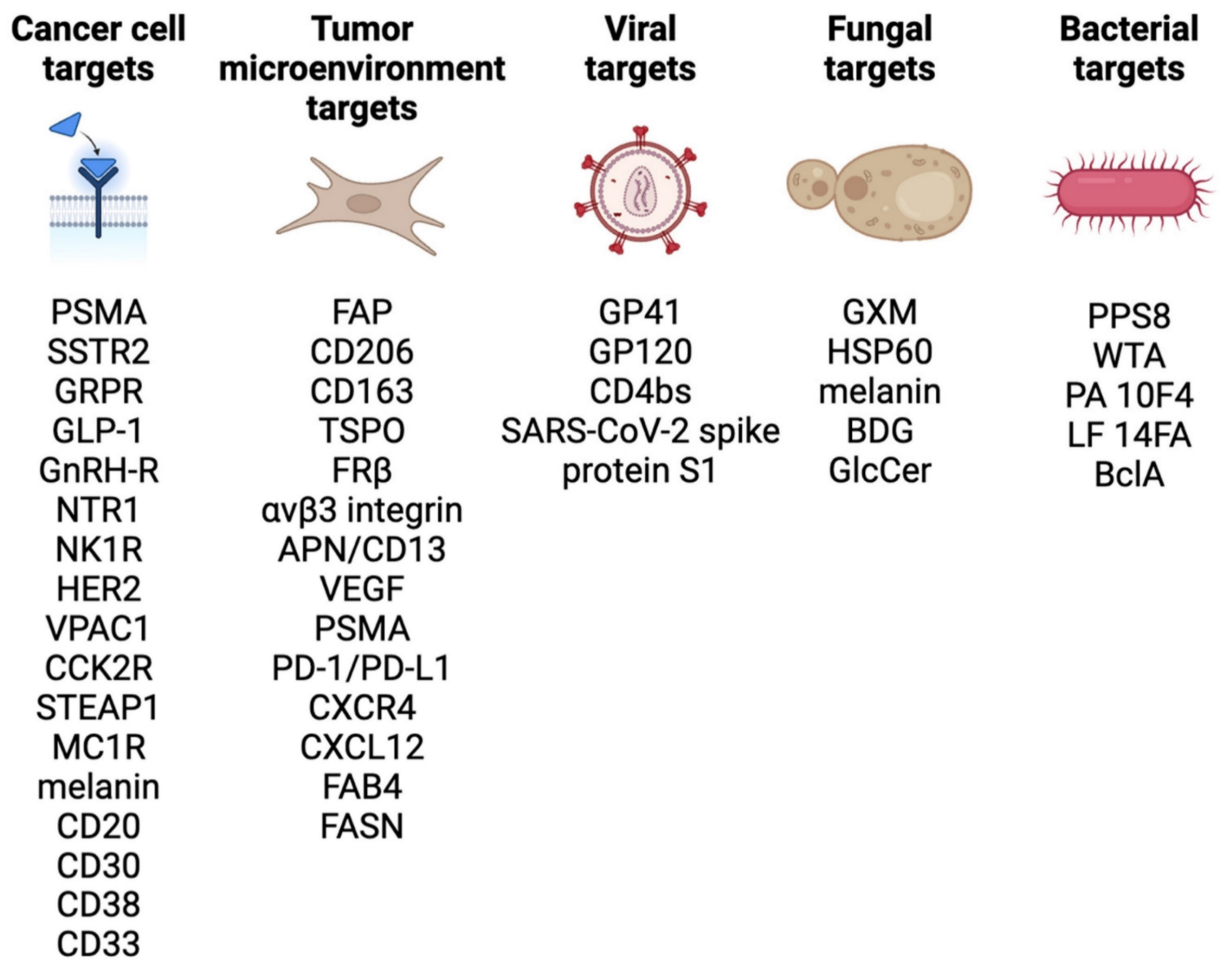
Select examples of cancer receptor, tumor microenvironment, viral, fungal, and bacterial theranostic targets. Figure made in BioRender.

A selection of theranostic targets under investigation is outlined in Figure 1. In general, targets should have high expression in pathological conditions (diseased tissue), and low expression in healthy conditions (healthy tissues) and the target should preferably be actively involved in a biochemical pathway of the pathological condition. Additionally, there should be a hypothesis regarding the theranostic target that involves a specific biological process or pathway. This approach will help elucidate the mechanism of action of the theranostic radiopharmaceutical and anticipate any changes in the disease microenvironment, so that the treatment can be optimized or potentially combined with other existing therapies. It may also be possible to enhance theranostic radiopharmaceutical efficacy and uptake at the site of the expressed target via strategies such as multi-receptor targeting, pharmacological upregulation of target receptors, and radiosensitization 27, so understanding the underlying biology and biochemistry are valuable when selecting a target for tracer development.

Crucially, some targets and vectors that are found to be well suited for imaging may not be suited for radionuclide therapy. For example, elevated glucose metabolism can be estimated using [^18^F]FDG and is a workhorse for diagnostic oncology, but unsuitable for therapy since elevated uptake will also occur in healthy tissue 28. Even if a suitable tracer is developed that possesses suitable pharmacokinetics and low off-target uptake, some targets may exhibit significant variation in expression among individuals, which could complicate therapeutic dosage. Additionally, established targets such as PSMA that are suitable for both imaging and therapy still face challenges due to the uptake of PSMA targeting vectors in healthy tissue including the kidneys, salivary glands, and lacrimal glands 29, leaving significant room for preclinical development and clinical translation of improved PSMA targeting vectors with reduced off-target uptake 30.

### 2.1. Theranostic targets explored in oncology

Some theranostic targets being investigated in oncology include PSMA in prostate cancer; SSTR2 in neuroendocrine tumors; gastrin-releasing peptide receptor (GRPR) expressed in multiple human malignancies; glucagon-like peptide 1 (GLP-1) receptor in insulinomas, gastrinomas, paragangliomas, medullary thyroid carcinomas, and pheochromocytomas; gonadotropin releasing hormone receptor (GnRH-R) in breast and prostate cancers; neurotensin receptor 1 (NTR1) in breast, prostate, colon, small cell lung cancers, pancreatic, and meningiomas; human epidermal growth factor receptor 2 (HER2) in breast and gastrointestinal cancers; neurokinin-1 receptor (NK1R) in gliomas; vasoactive intestinal peptide receptor 1 (VPAC1) in breast, prostate, ovarian, bladder, gastrointestinal, non-small cell lung, and pancreatic cancers; cholecystokinin-2 receptor (CCK2R) in medullary thyroid cancer; melanocortin receptor subtype 1 (MC1R) and melanin in melanoma; and the six transmembrane epithelial antigens of the prostate 1 (STEAP1) receptor in prostate cancer 31-35.

Theranostic targets for hematologic malignancies have also been extensively evaluated preclinically and in clinical trials, including CD20 for treating non-Hodgkin lymphoma, CD30 for treating Hodgkin lymphoma, CD38 for treating multiple myeloma, and CD33 for treating acute myeloid leukemia 36.

Some targeting vectors such as fibroblast activation protein inhibitors (FAPI) do not target cancer cells directly, but rather the surrounding fibroblasts that have proliferated and form a significant portion of the tumor microenvironment 37. FAP overexpression has been observed in at least 28 different tumor types, however tracer uptake in normal tissues including the salivary glands, thyroid, and oral mucosa could complicate therapeutic applications 9,38. Additional microenvironment targets include CD206, CD163, translocator protein (TSPO), and folate receptor beta (FRβ) on tumor-associated macrophages; α_v_β_3_ integrin, APN/CD13, vascular endothelial growth factor (VEGF), and PSMA on neo-angiogenic endothelial cells; programmed cell death protein-1 (PD-1), programmed death-ligand 1 (PD-L1), chemokine receptor type 4 and 12 (CXCR4/CXCL12) in tumor-infiltrating immune cells; and fatty acid-binding protein 4 (FAB4), and fatty acid synthase (FASN) on cancer-associated adipocytes 39.

### 2.2. Theranostic targets explored beyond oncology

Beyond oncology, there may be potential to image and treat refractory viral or antibiotic resistant bacterial infections and the associated therapeutic and immune responses 40. These targets include glycoprotein 41 (GP41), glycoprotein 120 (GP120), or the CD4-binding site (CD4bs) in the human immunodeficiency virus (HIV) 41,42; the SARS-CoV-2 spike protein subunit S1 43; wall teichoic acid (WTA) in the bacterial cell wall for *Staphylococcus aureus* prosthetic joint infections 40; pneumococcal capsular polysaccharide 8 (PPS8) in *Streptococcus pneumoniae* infection 44; the protective antigen (PA) 10F4 and lethal factor (LF) 14FA on *Bacillus anthracis* cells for anthrax infections 45; a collagen-like glycoprotein BclA found on *B. anthracis* spores 46; melanin, glucosylceramide (GlcCer), and glucuronoxylomannan (GXM) for *Cryptococcus neoformans* fungal infections 47,48; and heat shock protein 60 (HSP60) and beta-(1,3)-glucan (BDG) for *Candida albicans* and *C. neoformans* infections 49.

### 2.3. Hybrid theranostics

While this review focuses primarily on radiotheranostics where both the imaging and therapeutic vectors employ radionuclides, a hybrid approach can involve labeling a non-radioactive therapeutic targeting vector with a diagnostic imaging radionuclide to assess therapeutic potential. Cellular-based therapies such as CAR-T cell therapy can be tracked via molecular imaging with longer-lived PET radionuclides such as ^89^Zr 50,51, and non-radioactive therapeutic antibodies can be radiolabeled to evaluate their *in vivo* biodistribution 52. Depending on the targeting vector, this gives the potential to label a small amount of non-radioactive drug to perform initial biodistribution studies, increase the dose of non-radioactive drug if the biodistribution is favorable, and track subsequent treatment response with additional radiolabeled drug. Hybrid approaches may also be desirable for treatments involving diseases located in sensitive organs, such as glioblastoma, where it may be difficult to precisely deliver cytotoxic radionuclides to the tumor without significant off-target effects. Therefore, either a pure radiotheranostic approach or hybrid/integrated theranostic approach can be used to image and treat disease 12.

## 3. Theranostic radiopharmaceutical design and synthesis

### 3.1. Tracer selection and development

**Figure 2:**
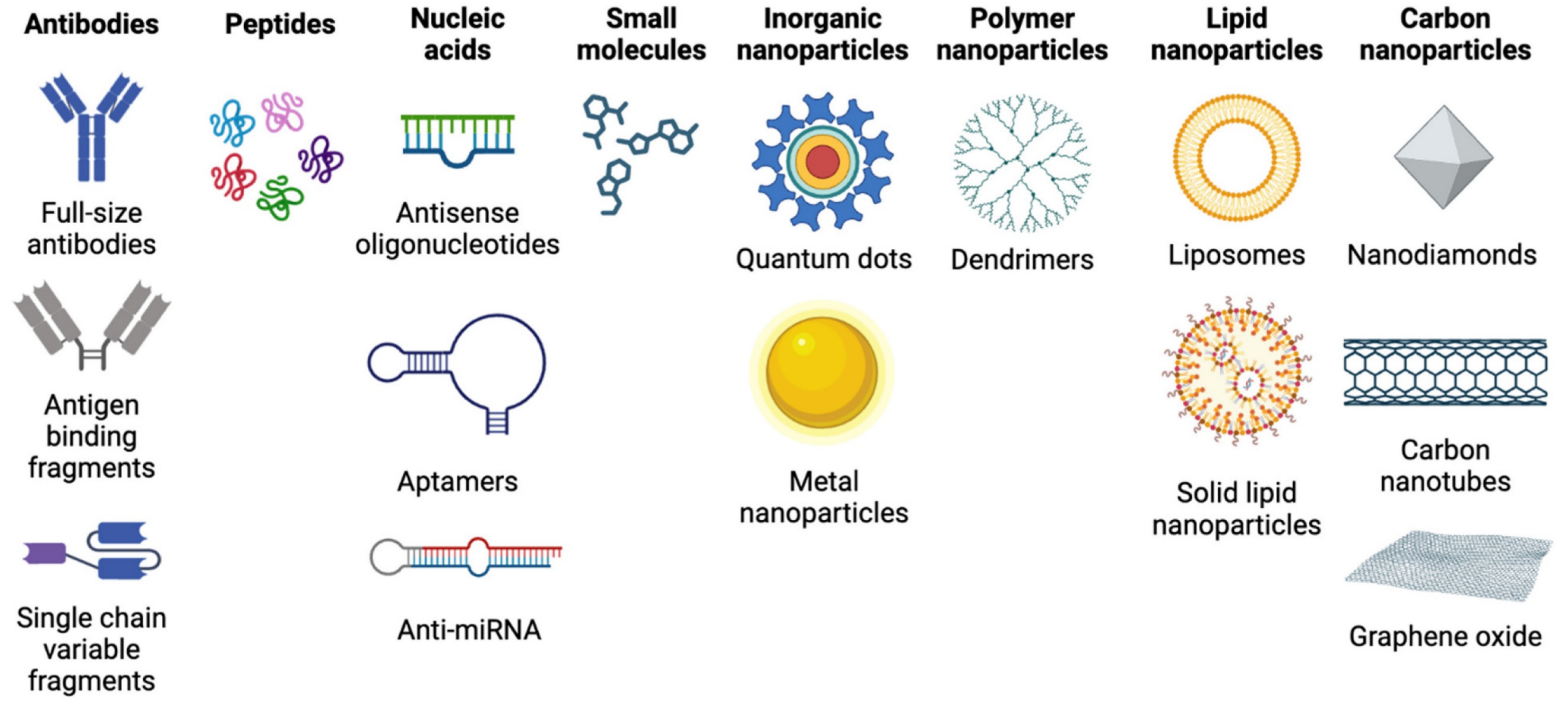
Examples of different radiopharmaceutical targeting vector categories. Figure made in BioRender.

Once a theranostic target has been identified, different types of targeting vectors can be considered for delivering the radionuclide. As depicted in Figure 2, targeting vectors typically fall into several categories that include antibodies, peptides, nucleic acids, small molecules, and nanoparticles 53.

Antibodies have a high affinity and specificity for antigens, allowing them to precisely deliver radionuclides to targets with high efficiency 54. However, full-size antibodies typically possess slow pharmacokinetics and low diffusivity within tumors, relegating their effective application to radionuclides with sufficiently long half-lives for target localization 53. They may also induce immunogenicity, particularly when targeted at cell surface markers, which can lead to undesirable side effects in patients 55, and result higher blood absorbed radioactive dose due to longer circulation times 56. Alternatively, antibody-derived scaffolds and fragments can be employed 57. These smaller antibody derivatives offer improved penetration into tissues, and typically possess serum half-lives of less than one hour, which can enable imaging at shorter time points post-injection 56,57. Additionally, antibodies can be employed for *in vivo* pretargeting strategies, where an antibody is first localized to tumors prior to injection of a radiolabeled small molecule that binds to the antibody 58. This strategy has potential to improve tumor-to-background uptake while reducing the off-target irradiation associated with conventional antibody approaches employing long-lived radionuclides 59.

Peptides possess advantages for radiopharmaceutical application including inexpensive synthesis, ease of radiolabeling, rapid tissue penetration, low toxicity and immunogenicity, and high affinity and specificity for target receptors 53. Peptides that have been explored for therapy include SSTRs, GRPRs, α_V_β_3_ integrin receptors, chemokine receptors 4 (CXCR4), melanocortin-1 receptors, and glucagon-like peptide 1 (GLP-1) receptors 60,61. Peptides need to attain a suitable balance between lipophilicity and hydrophilicity, so that the compound can cross the phospholipid bilayer of cells while maintaining sufficient solubility in water and avoiding membrane entrapment. This aspect of design impacts biodistribution, where more lipophilic peptides are eliminated via hepatobiliary excretion, while hydrophilic peptides are excreted via the kidneys 62. Limitations of peptides include a relatively short pharmacological half-life that can limit their imaging and therapeutic window, and renal absorption of hydrophilic peptides, which may result in nephrotoxicity limiting the tumor dose in peptide receptor radiotherapy 63. Several techniques exist that can overcome some of these drawbacks. Peptides that suffer from a short serum half-life can be modified by introducing D-amino acids and employing endcaps to reduce *in vivo* enzymatic degradation 62. Additionally, peptide cyclization can restrict conformational mobility and enhance receptor binding 64. To avoid interference with chelating groups used to sequester radiometals, chelators should be located a sufficient distance on the peptide from the receptor binding site, or spacer groups can be introduced between the chelator and peptide 62.

Nucleic acids used as theranostic vectors can include antisense oligonucleotides (ASO), which are short synthetic single stranded nucleic acids that pair with nucleic acid targets such as mRNA, and nucleic aptamers, which are oligonucleotides that can bind to a variety of targets including small molecules, proteins, nucleic acids, and cells 65. ASOs have been explored for imaging with ^99m^Tc, with anti-miRNA successfully radiolabeled with ^99m^Tc and tested in multiple tumor models 66. However, while nucleic acid-based vectors have a significant number of targets, they possess poor *in vivo* stability, making it difficult for them to achieve efficient delivery to their target. Modifications have been explored such as using anti-miRNA cell-penetrating peptides, which have demonstrated high specificity, sensitivity, cell uptake, and retention, and low cytotoxicity 67. Nucleic acid analogs such as antisense peptide nucleic acids (PNA) have been developed, along with PNA pretargeting approaches using affibodies, and shown to possesses improved uptake and efficacy 53. Nanoparticles have also been employed to improve ASO uptake, with liposomes and gold nanoparticles used to encapsulate ASOs for delivery 68.

Small molecules, which have a molecular weight less than 2000 Da, consist of most radiopharmaceuticals and possess advantages of rapid tumor penetration and clearance 54. Lipophilic small molecules can penetrate cell membranes rapidly, while hydrophilic small molecules take longer to reach cells as they pass through intercellular gaps. A variety of radiolabeled small molecules have been investigated for cancer therapy, especially PSMA-targeting molecules for prostate cancer. Small molecules are used in the pretargeting approach, where small molecules employing click chemistry are paired with larger molecules such as antibodies to overcome limitations associated with each individual vector 54. However, small molecules exhibiting rapid clearance may require more frequent dosing for therapy and will be limited to a shorter time window for effective imaging and therapy.

Nanoparticles with oncologic applications can be categorized in several groups: inorganic nanoparticles such as quantum dots, metal nanoparticles, iron oxide nanoparticles, silica nanoparticles, metal sulfide nanoparticles, and up conversion nanophosphors; polymer nanoparticles including core-shell dendrimers, and amphiphilic nanoparticles; lipid nanoparticles including liposomes, and solid lipid nanoparticles; and carbon-based nanoparticles, including carbon nanotubes, graphene oxide, and nanodiamonds. Nanoparticles possess a high surface area to volume ratio, a plethora of surface functionalization possibilities, and the ability to carry a significant amount of payload 69. They can accumulate at tumors via the enhanced permeability and retention (EPR) effect, entering tumors through fenestrations in leaky tumor blood vessels with restricted clearance routes due to poor lymphatic drainage 70, as well as transcytosis across vascular endothelium 71. However, limitations with relying exclusively on nanoparticles as a theranostic delivery vehicle include the reliance on the EPR effect, which exhibits high variability, and the involvement of the mononuclear phagocytic system (MPS), where cells of the MPS engulf injected nanoparticles, leading to immunosuppression or immunostimulation and potential toxicity 70. Further, nanoparticles may be non-specific to malignant tissue and exhibit off-target accumulation. To avoid some of these limitations with using nanoparticles alone, nanoparticles have been functionalized with peptides, antibodies, and aptamers to specifically bind with tumor receptors 53.

When developing a novel targeting vector, it can be useful to modify natural or previously developed compounds that have a demonstrated affinity for the target, with the aim of enhancing their target uptake and reducing off-target interactions with tissues around the body 72. Molecular mechanics, molecular docking, and pharmacophore modelling can be performed via computer-aided drug design (CADD) and machine learning techniques to accelerate development of promising compounds and assess the likelihood of radiopharmaceutical interaction with the target 73. As demonstrated with pretargeting techniques, it can be beneficial to integrate different classes of targeting vector to harness their unique strengths and reduce individual limitations. During development, the vector should be designed with the intention that it is stable in reaction solution and human serum to increase the likelihood that the radiopharmaceutical reaches its target before degradation. A higher stability also translates to a longer drug shelf life and improves flexibility for research, distribution, and potential clinical application. When selecting groups such as chelators to attach radionuclides to vectors, care should be taken to match established radiolabeling conditions, such as temperature and pH, for a given radionuclide and chelator with the anticipated stability of the targeting vector. For instance, chelators that require elevated reaction temperatures for extended periods of time may not be ideal for heat sensitive targeting vectors such as proteins and antibodies. Chelators should be carefully selected to ensure that they can stably sequester the radiometals of interest and achieve a sufficient molar activity during radiolabeling. Furthermore, the size, charge, and conformation of the resulting chelate complex can have a significant impact on radiopharmaceutical pharmacokinetics 74. This effect can be observed when changing radionuclides for a given chelator, or when using different chelators to sequester a given radionuclide, and result in a significantly altered compound biodistribution 75-77. As such, it may be useful to evaluate several chelator and radionuclide candidates in varying combinations with a given compound to assess which pair results in the most desirable pharmacokinetics.

Finally, with all delivery modalities, it is important to consider the choice of radionuclide so that the physical half-life of the radionuclide aligns with the pharmacological half-life of the targeting vector, and so the therapeutic radionuclide emissions have a suitable energy and pathlength for their anticipated delivery location within the disease microenvironment. Certain applications, such as imaging and therapy of neurological diseases such as glioblastoma, require special considerations over other targets such as the method of uptake through the blood-brain barrier (disrupted or undisrupted), the ability of the vector to diffuse within the tumor as well as penetrate infiltrative zone surrounding the tumor 78-80.

### 3.2. Radionuclide selection

**Figure 3:**
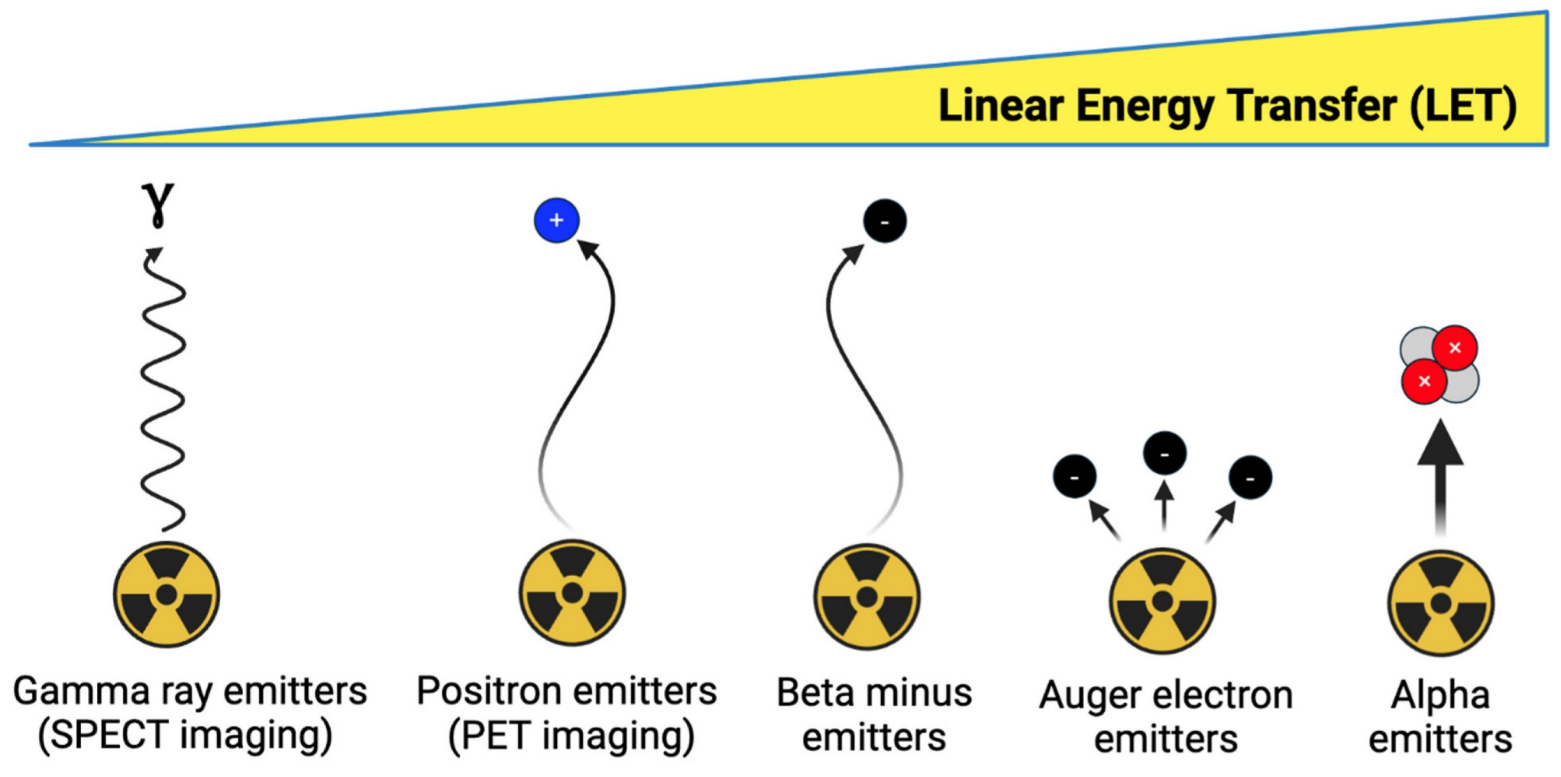
Overview of theranostic imaging and therapeutic radionuclide categories. Figure made in BioRender.

As depicted in Figure 3, imaging and therapeutic radionuclides possess different emission properties and should be selected to match the chemical and physical properties of their targeting vector 81. In general, targeting vectors with a longer biological half-life such as antibodies should initially be paired with an imaging radionuclide possessing a comparable physical half-life, so that the biodistribution of the radiopharmaceutical can be understood at extended timepoints 82. Vectors with a shorter biological half-life, such as small molecules, can be paired with radionuclides with a shorter half-life to minimize excess radioactive dose 81. However, longer-lived radionuclides can also be employed with shorter-lived vectors if they remain stably attached to the targeting vector and are excreted from the body in a timely manner 83.

When employing theranostic vectors with a single radiolabeling group, the diagnostic and therapeutic radionuclides should preferably possess similar or identical chemistries. This serves to streamline radiopharmaceutical synthesis steps and maintains similar coordination chemistry between the diagnostic and therapeutic compounds, reducing the likelihood of a significant difference in diagnostic and therapeutic compound biodistributions 83. Alternatively, if a targeting vector possesses multiple selective sites for radiolabeling, radionuclides of different chemistries can be employed, such as ^64^Cu with ^212^Pb, along with their stable Cu and Pb isotopic counterparts to maintain the same tracer biodistribution. Such a tracer possessing a NOTA chelator (chelates Cu but not Pb) and TCMC chelator (chelates Pb but not Cu) can be labeled with radioactive ^64^Cu and stable Pb for PET imaging, and conversely labeled with ^212^Pb and stable Cu for alpha particle therapy 24,25,84.

Besides chemical and physical considerations, diagnostic and therapeutic radionuclide selection should also be evaluated based on current and anticipated radionuclide availability, considering factors such as the number of facilities capable of producing the radionuclide, the supply of required target material, and distribution considerations particularly if the radionuclide is short-lived. Another important consideration is whether there are long-lived impurities (e.g. ^227^Ac) in radionuclide products that may have regulatory release limits and require extended sequestration of chemical and biological waste during preclinical studies or potential clinical trials 18.

The following subsections will outline some of the more promising therapeutic radionuclides and their corresponding diagnostic counterparts.

#### 3.2.1. Selecting a therapeutic radionuclide

**Figure 4:**
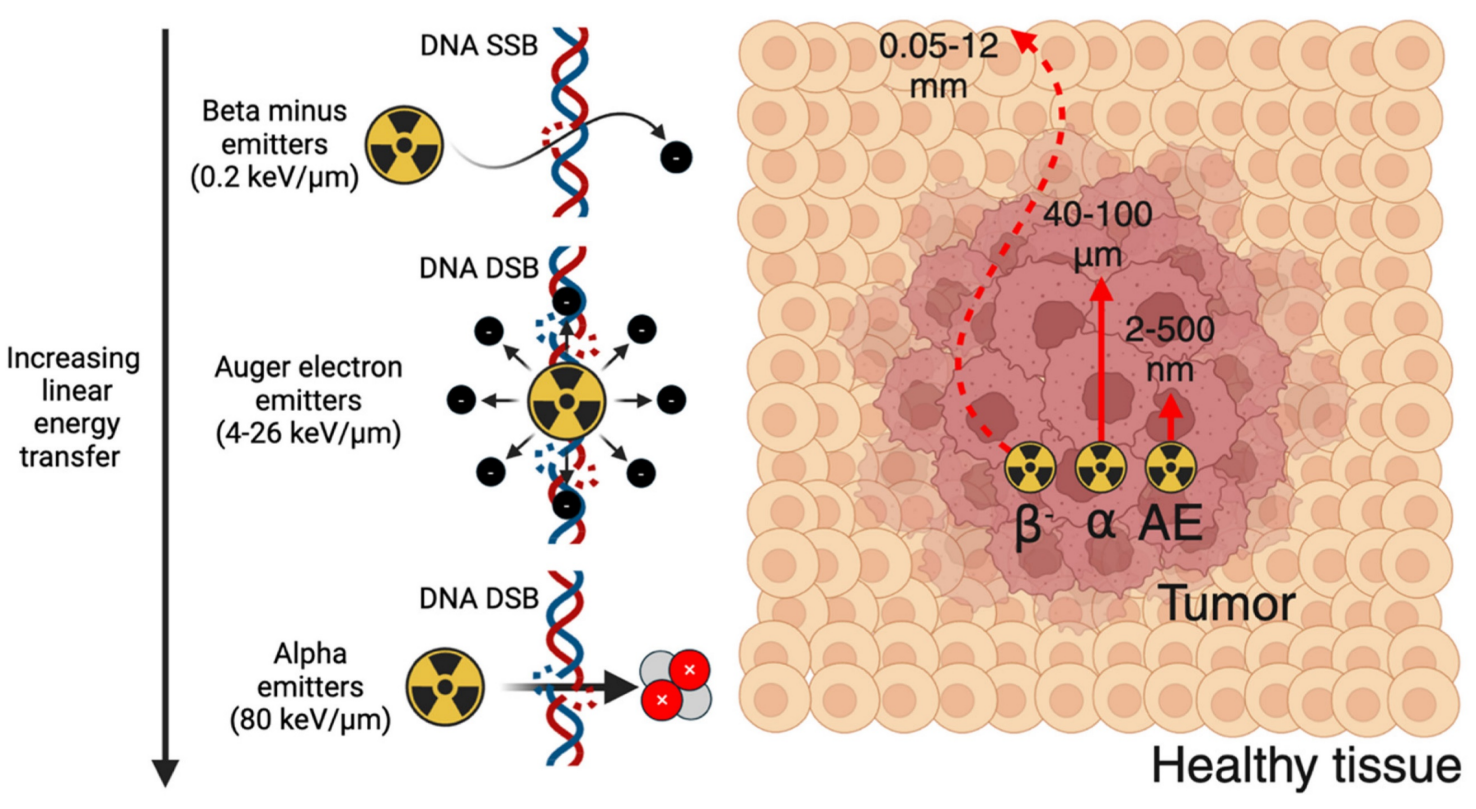
Summary of alpha, beta minus, and Auger electron therapeutic radionuclides and their primary DNA damage mechanism. Figure made in BioRender.

Outlined in Figure 4, therapeutic radionuclides used in nuclear medicine can be categorized as beta minus, alpha, and Auger electron (AE) emitters. Therapeutic radionuclide selection should be evaluated based on several factors, including physical half-life, chemistry, decay progeny, and emission characteristics 85. The physical half-life should be matched to the biological half-life of the targeting vector to optimally deposit radioactive dose at the disease site. The radionuclide should possess suitable chemistry so that it can be stably sequestered until it reaches its target, avoiding excess dose to the blood pool and healthy tissues. Radioactive decay progeny from the parent radionuclide, while potentially contributing additional therapeutic dose, should ideally remain localized to the disease site to avoid irradiating healthy tissue. Finally, radioactive emission characteristics have a significant impact on therapeutic outcome. Unless concurrent imaging is planned, the therapeutic radionuclide should produce few co-emitted x-rays or gamma-rays to reduce excess dose. For therapeutic particle emissions, a high linear energy transfer (LET), which represents the emission energy deposited per unit pathlength, is typically desired to maximize efficacy. Therapeutic radionuclides or their decay chain progeny often produce multiple emissions (β^-^, α, and AE), so this should be considered when determining the optimal therapeutic radionuclide for a given tracer.

Beta minus emitters possess a LET of 0.2 keV/μm, an emission energy per decay of 50-2300 keV, and a particle pathlength of 0.05-12 mm 86. Their relatively low LET results in significantly more DNA single-strand breaks (SSBs) relative to DNA double-strand breaks (DSBs). Owing to their longer electron emission pathlength, they are well-suited for irradiating larger metastases as opposed to micrometastases or circulating tumor cells 85. Significantly greater radiopharmaceutical activities are administered for therapy relative to PET or SPECT imaging, such as 7.4 GBq [^177^Lu]Lu-PSMA-671 administered every six weeks for four to six cycles 13. Some commonly used β^-^ emitters include ^177^Lu (t_1/2_ = 6.6 d), ^67^Cu (t_1/2_ = 2.6 d), ^90^Y (t_1/2_ = 2.7 d), ^131^I (t_1/2_ = 8 d), ^47^Sc (t_1/2_ = 3.4 d), ^161^Tb (t_1/2_ = 6.9 d), and ^188^Re (t_1/2_ = 17 h) 87.

Alpha emitters possess a significantly higher LET of 80 keV/μm, an emission energy of 5-9 MeV, and a particle pathlength of 40-100 μm 86. The high LET emissions of alpha emitters result in a dense ionization track that induces significant DNA DSBs within affected cells, and results in cell death independent of oxygenation or the cell cycle 88. This makes alpha emitters much more cytotoxic compared to β^-^ emitters and well suited for eradicating micrometastases and circulating tumor cells. Their potency results in significantly lower activities being used relative to β^-^ emitters, with clinical dose ranges such as 10-120 kBq/kg in a single or multi-dose regimen explored for [^225^Ac]Ac-FPI-1434 targeting IGF-1R, and four cycles of 2.5 MBq/kg for [^212^Pb]Pb-DOTAMTATE targeting SSTR-2 89. As such, many clinical trials utilizing alpha emitters have been initiated with the aim of exploiting the fundamental advantages of alpha emitters to improve upon β^-^ therapy. Radionuclides explored for alpha therapy include ^225^Ac (t_1/2_ = 9.9 d), ^212^Pb (t_1/2_ = 10.6 h), ^211^At (t_1/2_ = 7.2 h), ^227^Th (t_1/2_ = 18.7 d), ^224^Ra (t_1/2_ = 3.6 d), ^223^Ra (t_1/2_ = 11.4 d), and ^149^Tb (t_1/2_ = 4.1 h) 87.

Auger electron emitters possess a LET of 4-26 keV/μm, an emission energy of 0.2-200 keV, and a particle pathlength of 2-500 nm 86. This limits their direct therapeutic effects to a single cell, however they also have potential to be highly cytotoxic through inducing DNA DSBs if delivered close to sensitive cellular compartments 90. Radionuclides that have been considered for AE therapy include ^99m^Tc (t_1/2_ = 6 h), ^123^I (t_1/2_ = 13.2 h), ^67^Ga (t_1/2_ = 3.3 d), ^125^I (t_1/2_ = 59 d), ^191^Pt (2.8 d), ^201^Tl (3.0 d), ^119^Sb (1.6 d), ^111^In (2.8 d), and ^135^La (19.5 h) 90-92.

The availability of β^-^ emitters such as ^177^Lu is currently better compared to most alpha or AE radionuclides since β^-^ emitters are more established in clinical nuclear medicine 93. However, significant efforts are underway to improve alpha emitter availability 18,22, and more AE emitters are under production for preclinical investigation 92.

Beyond these three traditional categories of therapeutic particle-emitting radionuclides, positron emitters have also been recognized as a potentially useful therapeutic radionuclide. Monte Carlo simulations of positron and electron emissions showed that for a given particle emission energy, induction of DNA SSBs and DSBs and linear energy transfer were all higher for positrons relative to electrons, across the entire energy range 94. While the significant activities required for positron therapy relative to PET imaging (potentially activities of a similar magnitude to β^-^ therapy) would result in shielding concerns owing to the highly penetrating 511 keV annihilation photons, the low cost and high availability of positron emitters with established targeting ligands may justify exploring their therapeutic feasibility 94.

#### 3.2.2. Selecting a diagnostic radionuclide

Ideally, theranostic imaging surrogates should possess a comparable half-life and similar chemistry to their therapeutic counterparts. If no suitable imaging isotopes of the same element are available, chemically similar elements can be used 83. While ^68^Ga and ^18^F are readily available and widely used as PET imaging radionuclides for radionuclide therapy, they possess significantly shorter half-lives and different chemistries relative to most therapeutic radionuclides, which makes them unideal imaging surrogates for theranostics 95,96.

**Figure 5:**
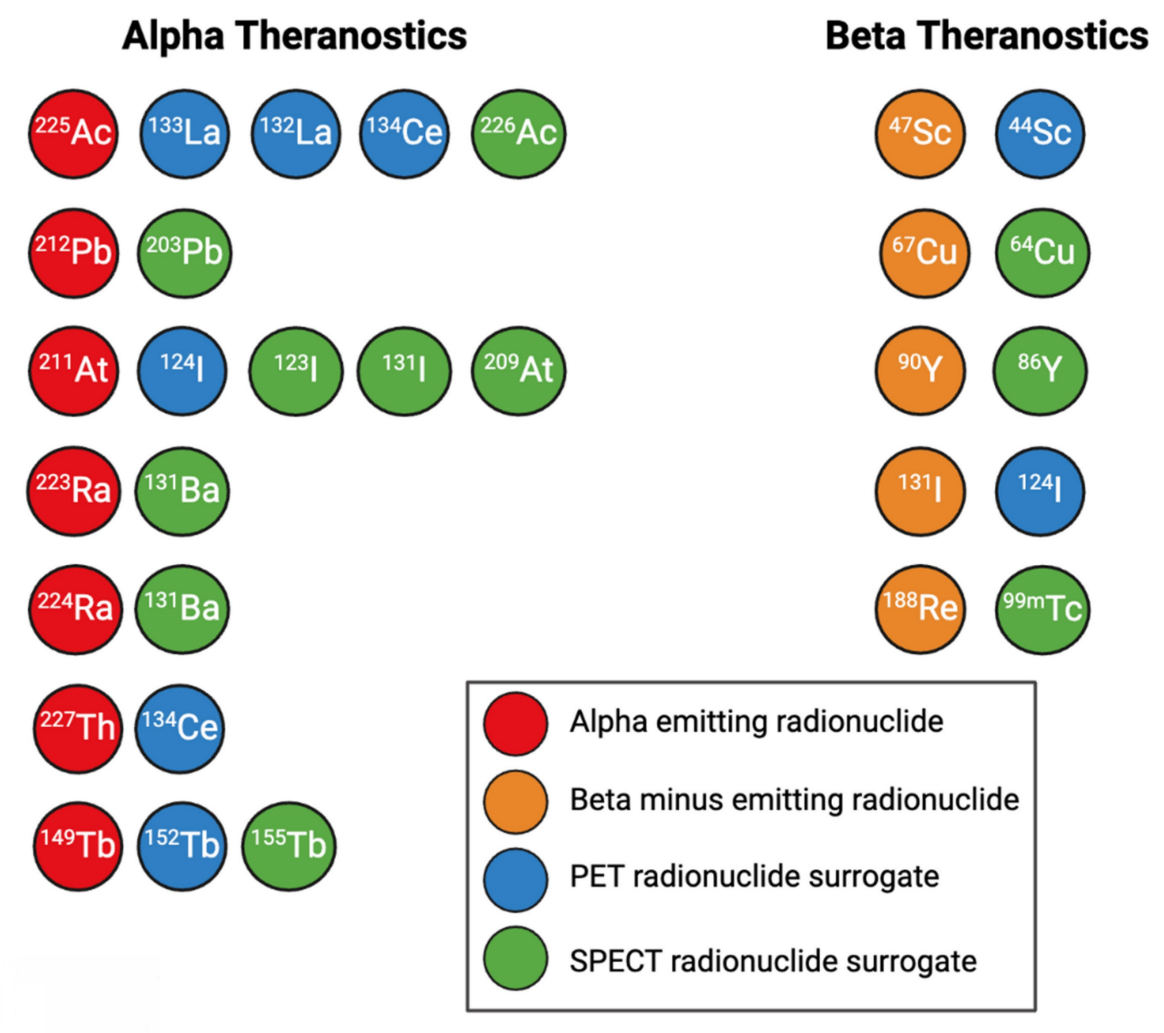
A selection of commonly used alpha and beta therapeutic radionuclides and their PET and SPECT imaging surrogates. Figure made in BioRender.

PET imaging radionuclides should ideally have a low positron emission energy, which corresponds to a higher imaging spatial resolution, a high positron branching ratio, and few co-emissions that result in excess radioactive dose. SPECT imaging radionuclides should ideally possess lower energy gamma rays within the optimal energy window of scanner collimators, and few co-emissions to reduce noise 83,97. A summary of diagnostic PET and SPECT imaging surrogates for alpha and β^-^ emitters is shown in Figure 5.

Some surrogates for β^-^ emitters include ^44^Sc for PET imaging of ^47^Sc 98; ^64^Cu for PET imaging of ^67^Cu 76,99, ^86^Y for PET imaging of ^90^Y 11, ^124^I for PET imaging of ^131^I 100, and ^99m^Tc for SPECT imaging of ^188^Re 101. Figure 10 gives an example of the similar biodistributions observed with ^64^Cu PET imaging and ^67^Cu SPECT imaging, where ^64^Cu and ^67^Cu were labeled to the same targeting vector 15.

For alpha particle emitting radionuclides, diagnostic surrogates include ^133^La 91,102-105, ^132^La 106-108, and ^134^Ce/^134^La for PET imaging of ^225^Ac and ^227^Th 109; ^226^Ac for SPECT imaging of ^225^Ac 110-112; ^203^Pb for SPECT imaging of ^212^Pb 113-118; ^123^I, ^131^I, and ^209^At for SPECT imaging of ^211^At 83,119-121; ^124^I for PET imaging of ^211^At 83,100; ^131^Ba for SPECT imaging of ^223/224^Ra 122; ^155^Tb for SPECT imaging of ^149^Tb, and ^152^Tb for PET imaging of ^149^Tb 83. Figure 11 showcases the similar biodistributions obtained from ^203^Pb/^212^Pb SPECT imaging 123.

Many AE emitters such as ^99m^Tc, ^67^Ga, ^123^I, and ^111^In possess suitable gamma emissions for SPECT imaging 124. For AE emitters such as ^135^La without sufficient intensity co-emissions for imaging, diagnostic surrogates such as ^133^La can be employed 91.

While availability has been improving for imaging surrogate radionuclides, production remains far below that of ^99m^Tc, ^18^F, and ^68^Ga. Additionally, some imaging surrogates such as ^152^Tb, ^209^At, and ^226^Ac are only capable of being produced at a handful of specialized facilities, which significantly limits their potential applications 83.

### 3.3. Radiopharmaceutical synthesis

For a radiopharmaceutical synthesis, typical components include the precursor molecule to be labeled, the radionuclide in a form amenable for radiolabeling, a buffer to maintain appropriate pH, a scavenging agent to reduce radiolysis, and a suitable diluent for subsequent radiopharmaceutical application 125. Synthesis should be performed in the simplest manner possible, ideally as a one pot reaction, with the fewest number of post-labeling purification steps required. Reactions are preferably performed at room temperature to reduce potential degradation, which may be necessary for larger heat sensitive targeting vectors 126. Radiolabeling efficiency, the ability for a chelator to form its radionuclide complex, should be as high as possible to minimize the required radionuclide activity and avoid further solid phase extraction (SPE) or lengthy HPLC purification. This is particularly important for vectors that exhibit reduced *in vitro* or *in vivo* stability, and with vectors labeled with short half-life radionuclides. Reducing unnecessary steps also streamlines *in vitro* and *in vivo* preclinical experiments, which may require significant amounts of time, and can become more difficult for research groups as the time required for radiopharmaceutical synthesis increases.

One of the most important parameters to report in the synthesis of radiopharmaceuticals is the molar activity (A_m_) or specific activity (A_s_). The molar and specific activity are a measure of the amount of radioactivity present per mole (A_m_) or mass (A_s_) of compound, respectively, in a solution containing a radiotracer. These and other reported parameters should adhere to the nomenclature guidelines outlined by Coenen *et al.* 127. The total amount of compound present is the sum of the quantity of radioactive compound (M_A*_) plus the quantity of the corresponding isotopically stable compound (M_A_), as depicted in Eq. 1 128. Generally, it is advantageous to synthesize radiopharmaceuticals with the highest A_m_ practically achievable 129.


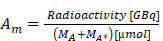

((Eq.1)

**Figure 6:**
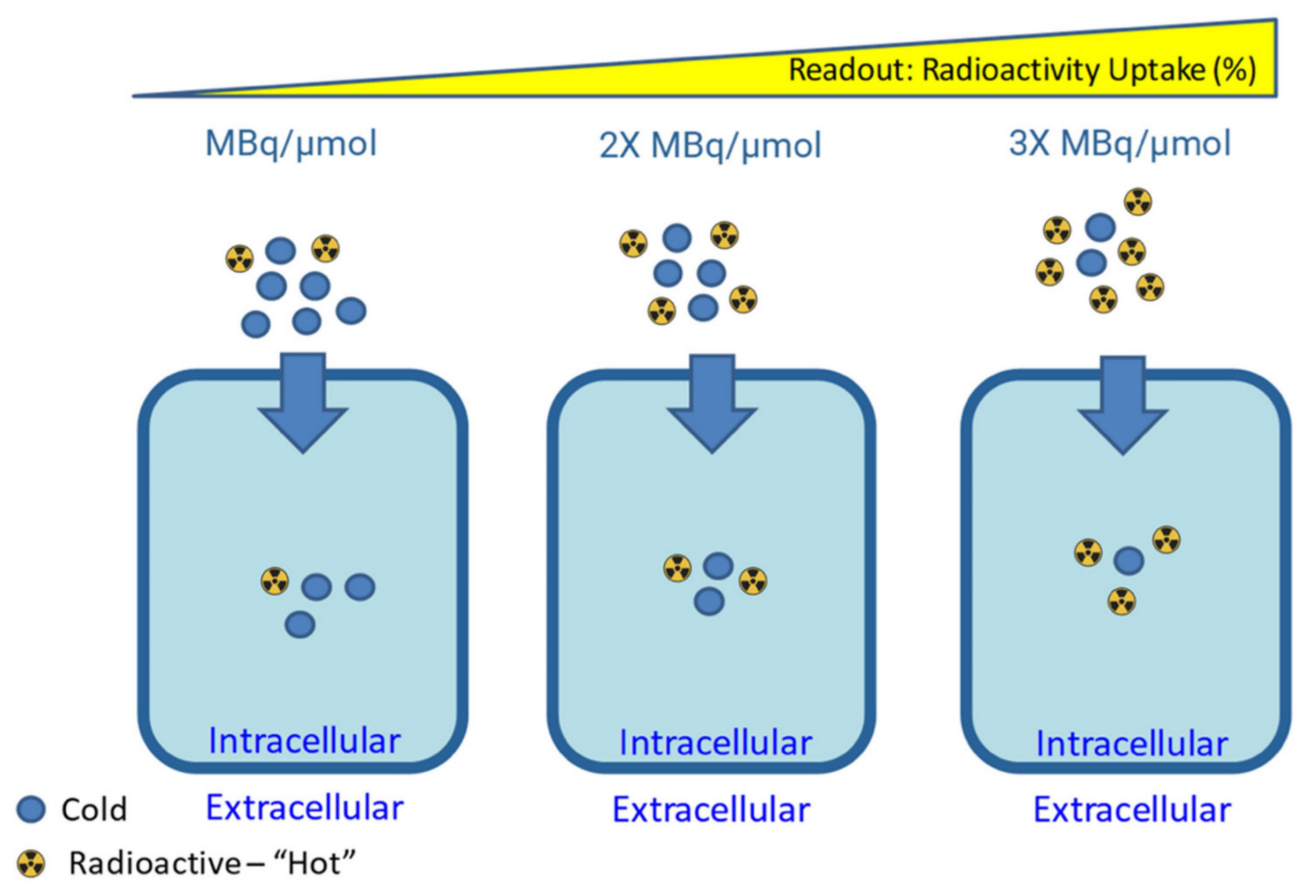
Canonical impact of molar activity on radioactivity uptake for a targeting vector unaffected by secondary biological effects. Figure made in BioRender.

A useful derivative of molar/specific activity is the *apparent* molar/specific activity. This expands on Eq. 1 by adding more terms to the denominator by considering the presence of any other impurities in the radiopharmaceutical formulation, including the remaining precursor, radiolabeled impurities or other isotopically stable impurities which may compete with and affect the radiotracer performance *in vivo*. As depicted in Figure 6, high molar activity can be crucial for low density targets such as neuroreceptors, as it reduces competition for binding sites with non-radioactive tracer mass 130. On the other hand, some targeting vectors may require reduced molar activity to achieve optimal uptake during *in vitro* and *in vivo* experiments 131. This could be due to the compound exerting biological effects that increase target expression or alter targeting vector clearance, and the presence of a “sink” outside the target tissue, where the compound is metabolized or undergoes non-specific binding 130,131.

**Figure 7:**
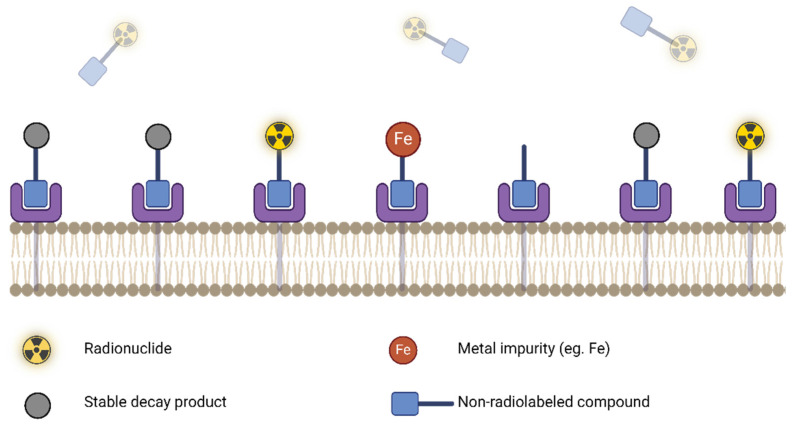
Reduced radiotracer uptake due to the presence of competing stable impurities (low apparent molar activity). Figure made in BioRender.

The molar activity is time dependent and accurate/precise reporting requires sensitive and robust characterization (UV-Vis, HPLC) with every production. Inconsistency in this reporting can make cross-institutional data sharing challenging and can complicate the interpretation of results. High apparent molar activity can be achieved by taking steps to remove contaminants that could bind or degrade the precursor. Precursors and reagents should be ultra-pure, trace metal grade, and contain minimal stable isotope impurities of the radionuclide in use. This is important, since stable isotopes can outcompete radionuclides of the same element, resulting in poor radiolabeling yields 95. Figure 7 demonstrates how even in the case of high target receptor density and high targeting compound uptake, a low molar activity can nonetheless result in low effective uptake of the radiolabeled compound due to the presence of stable competing contaminants.

During synthesis, it is recommended to use PEEK or Teflon materials (unreactive), avoid any contact with metal surfaces if working with radiometals, and line the inside of hot cells and fume hoods to prevent corrosion and potential product contamination 95. If there are issues with the synthesis that cannot be explained by reaction conditions, a thorough examination of synthesis reagents can be performed using elemental analysis equipment such as inductively coupled plasma optical emission spectroscopy (ICP-OES) or inductively-coupled plasma mass spectroscopy (ICP-MS) 95. This analysis can include the cyclotron or nuclear reactor-derived radionuclide product and the reagents used to synthesize and purify the radionuclide from its irradiated target material.

If radiopharmaceutical stability is an issue due to inherent compound degradation or radiolysis from high activity concentrations, stabilizers such as ascorbic acid, gentisic acid, or ethanol can be added to reduce radiolysis and increase product shelf life 125.

While preclinical radiopharmaceutical synthesis is often initially performed manually, automation should be considered from the start of development, especially if preclinical data shows promise for clinical translation.

### 3.4. Automation and quality control

**Figure 8:**
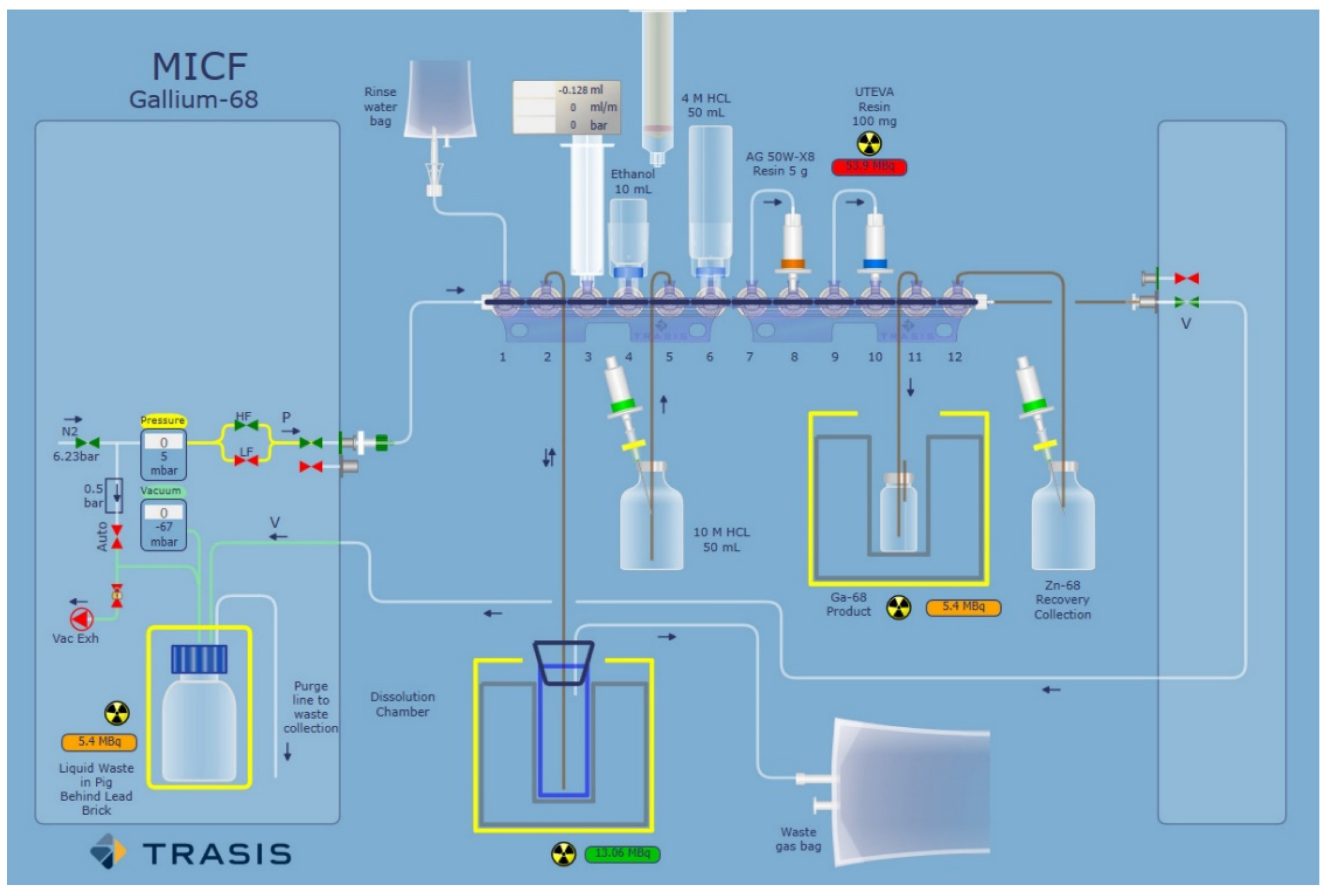
Example of a TRASIS all-in-one graphical user interface used for radionuclide purification. Adapted with permission from 96, copyright 2020 Elsevier.

After successful manual synthesis of a theranostic radiopharmaceutical, automation should be considered to save time, enhance reproducibility, and prepare for potential clinical applications. Larger preclinical experiments may also warrant automation, especially if the manual synthesis contains labor-intensive steps. A flexible automated synthesis unit (ASU) with demonstrated reliability (few mechanical failures), and precise control should be employed. For instance, a cassette-based ASU designed to facilitate good manufacturing practices (GMP) compliance will expedite development and simplify establishing the process on other synthesis modules 95. Further, as depicted in Figure 8, it is beneficial if the ASU software has an easy-to-use graphical user interface (GUI), where the GUI supports both process development and active readout of parameters during production runs. Quality control techniques involving radio-TLC or radio-HPLC should be simplified to be conducive to radiopharmacy production schedules and reduce costs. Radio-TLC methods should clearly distinguish free radionuclide from the labeled compound. To better quantify any present impurities, a two radio-TLC system should be used to improve analytical certainty. Radio-HPLC techniques should clearly separate any impurities from the radiolabeled product in a relatively short time. For both diagnostic and therapeutic radionuclides, the reaction should be demonstrated to be scalable for multiple patient doses, which may require adjusting product composition if radiolysis is observed at higher activities 132. Product specifications that require assessment include appearance, pH, radiochemical purity/yield, radionuclidic purity, radioactivity concentration, tracer identity, molar activity, chemical purity, residual solvents, bacterial endotoxins, sterility, and stability 133. The duration of product stability analysis will depend on tracer properties and the half-life of the radionuclide. For instance, a small molecule labeled with ^68^Ga may not require stability analysis beyond several hours, whereas an antibody labeled with ^89^Zr or ^225^Ac may require analysis at extended timepoints post-labeling. Product solutions can be analyzed via radio-TLC to assess radionuclide incorporation and radio-HPLC to assess potential compound degradation in the reaction solution over time 134. Once quality control procedures have been established along with reliable synthesis, the radiopharmaceutical can be used for preclinical experiments.

## 4. Preclinical theranostic evaluation

**Figure 9:**
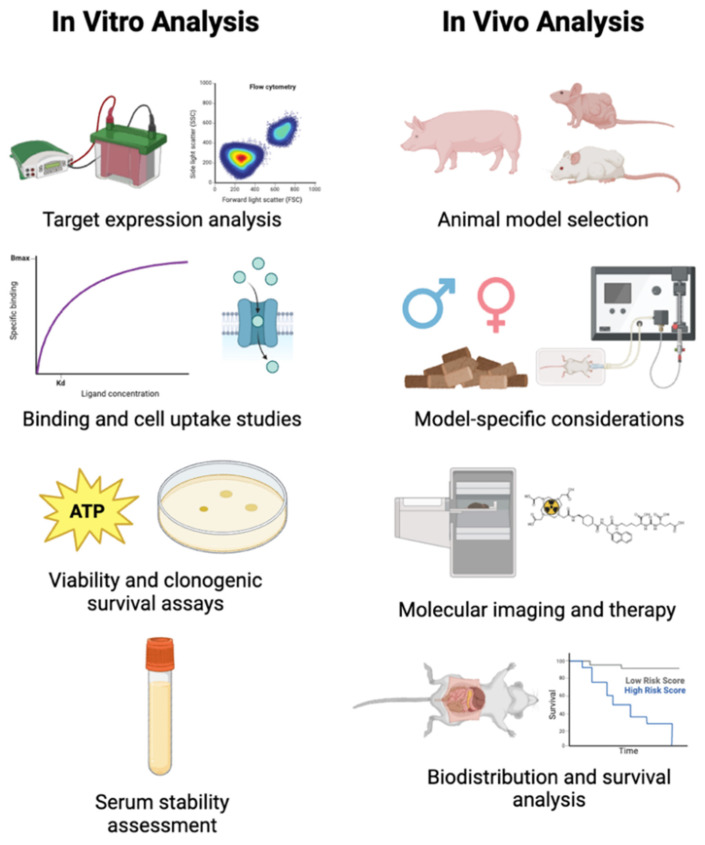
Aspects of preclinical theranostic radiopharmaceutical development. Figure made in BioRender.

**Figure 10:**
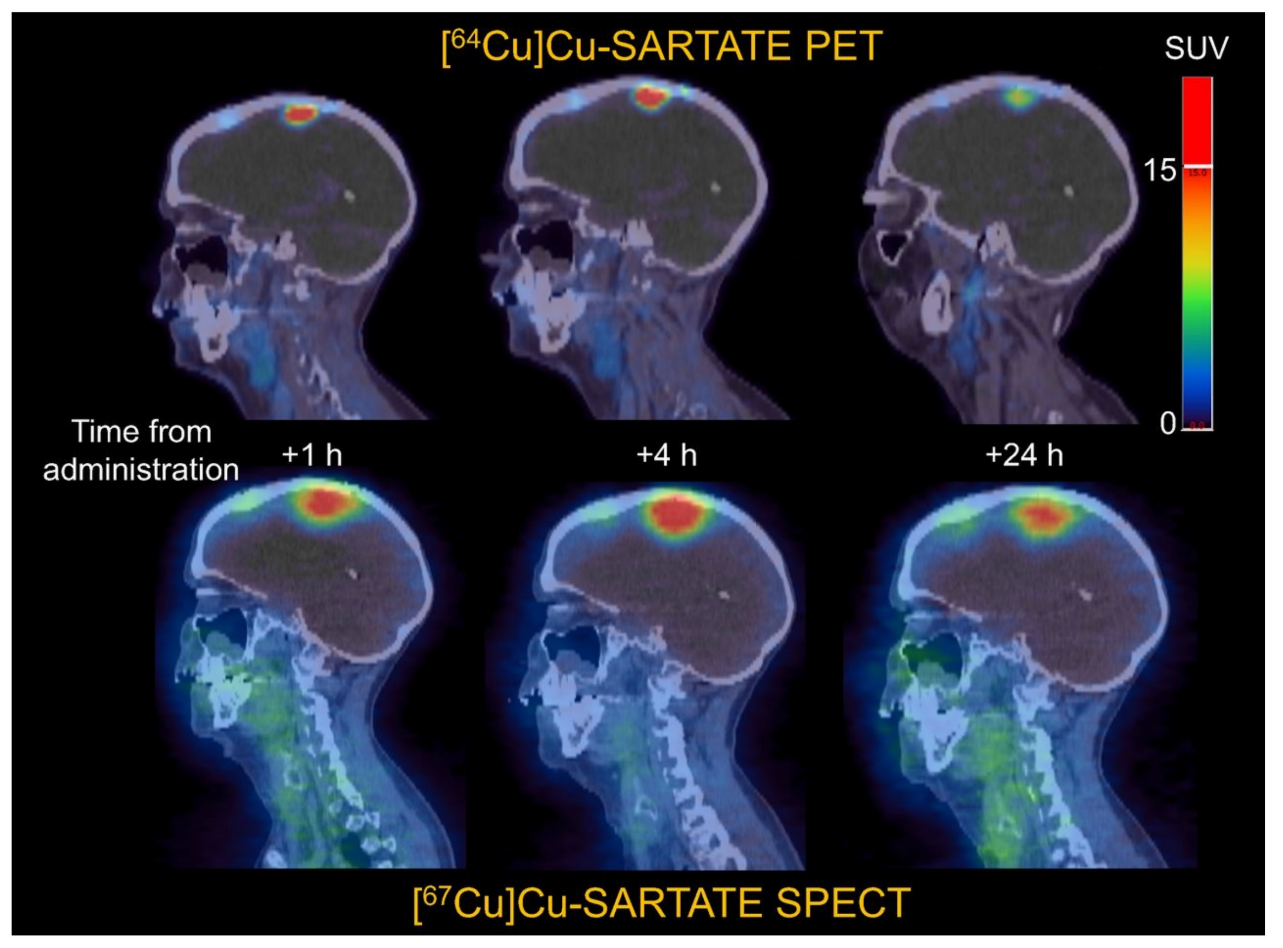
Comparison of clinical ^64^Cu PET imaging and ^67^Cu SPECT imaging, highlighting how changing the radionuclide from ^64^Cu to ^67^Cu does not alter tumor targeting. Adapted with permission from 15, copyright 2022 SNMMI.

**Figure 11:**
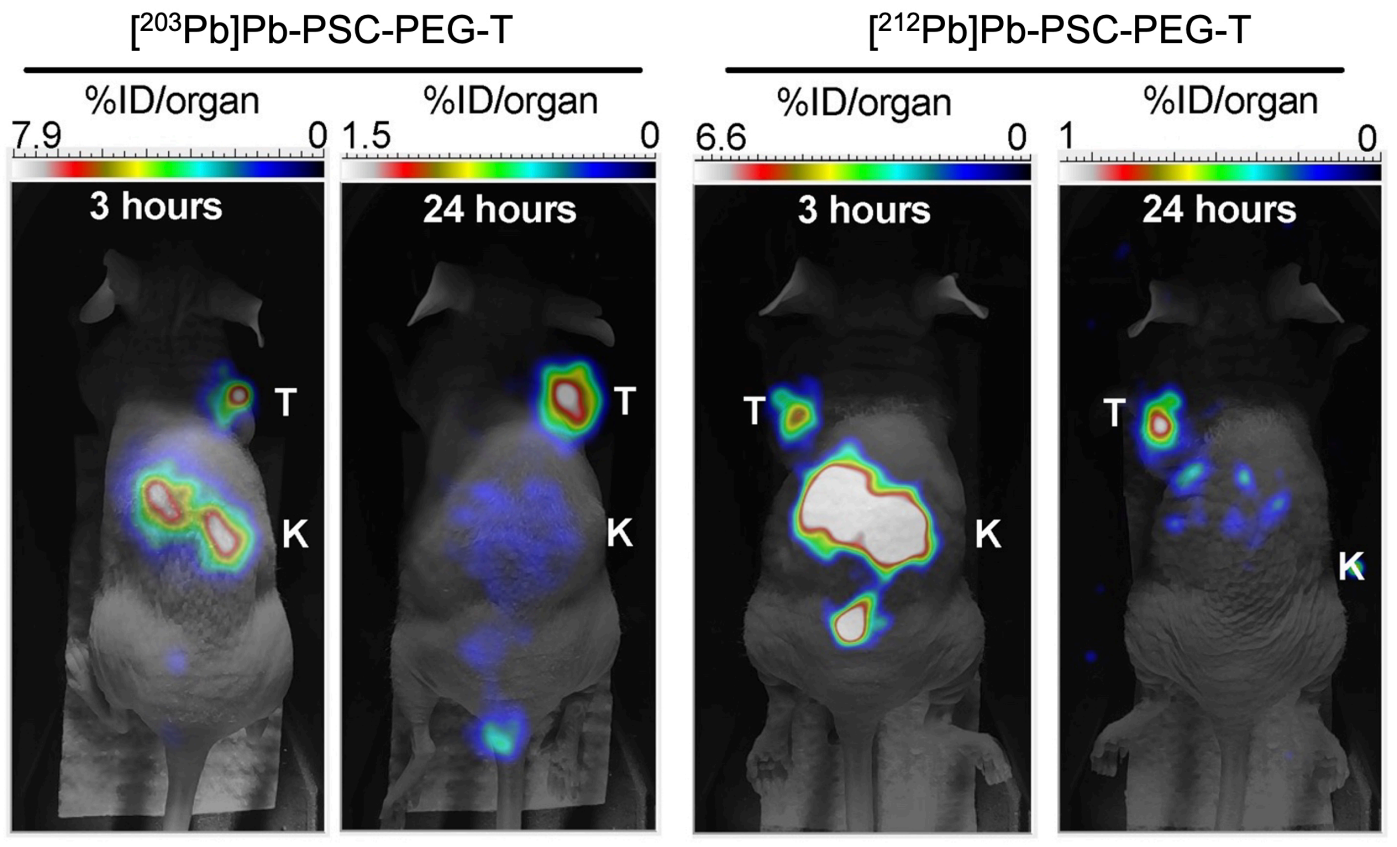
Preclinical comparison of ^203^Pb and ^212^Pb SPECT imaging, demonstrating the similar biodistribution and tumor-targeting properties of the ^203^Pb imaging and ^212^Pb therapeutic compounds. Adapted with permission from 123, copyright 2023 MDPI.

There are several general steps in preclinical theranostic radiopharmaceutical development, as outlined in Figure 9. First, radiopharmaceuticals should be evaluated in cell culture to determine if they are specific for the intended target. Then, a suitable animal model should be selected for initial imaging and pharmacokinetic analysis. If these results indicate a favorable compound biodistribution, then combined imaging and therapy studies can be performed.

### 4.1. *In vitro* radiopharmaceutical analysis

After quality control and stability analysis, *in vitro* cell studies with the theranostic radiopharmaceutical should be performed prior to animal experiments. The purpose of *in vitro* analysis is to assess the binding affinity of the radiopharmaceutical, perform competitive binding studies to validate radiopharmaceutical binding, assess serum stability, and perform cell uptake studies to assess radiopharmaceutical uptake and cell survival to rule out any acute toxicity.

Several cell lines should be selected that are known to express the target of interest, and at least one other cell line that does not express the target of interest can be selected as a negative control. For example, when selecting a prostate cancel model for PSMA, LNCaP and CWR22Rv1 prostate cancer cells endogenously exhibit significant PSMA expression, while PC3 and DU145 prostate cancer cells do not express PSMA 135. Analysis can be performed on cell lines to verify the presence of the target of interest using techniques such as western blotting or flow cytometry 136. The half-maximal inhibitory concentration (IC_50_) should be determined to assess the potency of leading compounds 137. This can be evaluated using a cell line positive for the target of interest, the compounds of interest along with a standard with known target affinity, a radiotracer with known affinity for the target.

To determine cell uptake, cells should be cultured and plated according to established protocols, and the radiotracer should be incubated with the cells. At given time points after applying radioactivity, the cells should be washed to remove residual unbonded radioactivity. Cells can then be removed, and their radioactivity quantified on a gamma counter 137. These cell uptake experiments can be performed with both diagnostic and therapeutic radionuclides to assess any variations in cell radioactivity uptake. Cell uptake studies should also assess the internalization of the radiopharmaceutical using a technique such as a glycine wash, which removes membrane-bound compound 138. This is particularly important for radiopharmaceuticals with alpha or AE emitting radionuclides since internalization of the alpha or AE emitters may bring their cytotoxic emissions closer to sensitive cellular compartments. It may also be useful to examine the subcellular radiopharmaceutical distribution. Localization techniques include subcellular fractionation, micro-autoradiography, fluorescence imaging, X-ray fluorescence microscopy, laser ablation-ICP-MS, or ion beam analysis 139. For more reliable results, cell radioactivity uptake should be normalized to the number of cells and radiopharmaceutical specific activity, using techniques such as a bicinchoninic acid (BCA) assay 138. If additional steps are performed, such as a glycine wash to assess internalization, it is important to separately determine the protein content for cells subjected to the altered conditions. Incubations can also be performed in a hypoxia chamber to assess the impact of varying oxygen levels on compound uptake, and mimic *in vivo* conditions found within tumors and tissues with naturally lower oxygenation 140.

The impact of molar activity should be assessed throughout *in vitro* cell uptake experiments to determine if the mass of non-radioactive precursor has any effect on the radiopharmaceutical uptake 130.

Preclinical therapeutic experiments can be performed to assess if the radiopharmaceutical exerts a cytotoxic effect on cells. Therapeutic radiopharmaceuticals can be administered to cells in varying doses to assess the magnitude of a dose-dependent cytotoxicity effect. Cell viability assays can be performed to assess if cells are still metabolically active after radiopharmaceutical application, and clonogenic survival assays can determine if cells still possess colony forming potential after therapy 141.

The radiopharmaceutical should also be incubated in serum at 37 ºC for timepoints longer than the expected *in vivo* residence time. This is valuable data to acquire that can inform subsequent *in vivo* biodistribution and imaging experiments. Serum stability analysis can be performed with radio-TLC to assess radiolabeling stability of the radionuclide with its carrier, and radio-HPLC can be used to assess the stability of the compound in serum over time. If radio-HPLC is employed, the serum incubation mixture should be diluted significantly prior to injection to avoid excessive serum protein buildup on the HPLC guard column, and HPLC pressure should be regularly monitored.

### 4.2. Selecting a suitable *in vivo* model

Once compound binding characteristics, cell uptake, and other tracer properties have been evaluated *in vitro*, *in vivo* experiments should be performed to assess the theranostic potential of the radiopharmaceutical. Animal model selection should evaluate established disease models, ethical considerations, and economic feasibility, which preferably achieves a sufficient sample size to confer statistical significance 142. The animal source should be considered, including its commercial availability, where it is developed/maintained, or alternatively if a model can be developed locally using reproducible non-proprietary techniques. The four “R” principles for animal experiments of replacement, reduction, refinement, and responsibility should be considered at all stages of preclinical development 143. Rodents such as mice are often used for cancer research, owing to their commercial availability, well-established disease models, and favorable economics. However, limitations include a small blood volume that reduces permissible sampling size, and smaller organs which hinder imaging studies that would benefit from higher resolution, such as neuroimaging. These limitations can be overcome by using larger animals such as pigs, however at significantly greater expense 144. For theranostic tracers, using an immune competent versus an immunocompromised model is an important consideration. For instance, head and squamous cell carcinoma (HNSCC) can be studied in immunocompetent mice using a chemically induced model, where carcinogens are used for tumor induction; a syngeneic transplant or allograft model, where homologous tumor cell lines are injected into mice to avoid rejection; or a genetically engineered model, where tumors can closely mimic the heterogeneity and histopathological features of human tumors 145-147. HNSCC can be studied in immunocompromised mice using a nude mouse model, which lack a thymus and results in T-cell deficiency; a SCID model, which has T- and B-lymphocyte defects; a NOD/SCID mouse model, which lacks both functional lymphoid cells and natural killer cells; or an NSG model, which is even more immunodeficient than NOD/SCID mice due to deletion of the IL-2 receptor common gamma chain 147. While immunocompromised animal models may possess lower rates of tumor rejection for imaging and therapeutic studies, they lack the ability to mount a significant antitumor immune response. While this may not be a significant concern for diagnostic SPECT or PET imaging, this could complicate the results of targeted radionuclide therapy. It is known that the destruction of cancer cells via TRT may release tumor associated antigens and other factors that can induce a significant antitumor immune response 148,149. Additionally, recent studies have found synergistic effects between immune checkpoint inhibitors and TRT 150, motivating the use of both immunocompetent and immunocompromised mice to study any differences in antitumor response. In some instances, radiopharmaceuticals need to be tested in a transgenic or patient-derived xenograft (PDX) model that replicates human disease in animals 151. Some transgenic disease models for common disease targets are outlined in Table 1 152-155.

Further, the sex of the animals should be considered. Cancers that are experienced by one sex, such as ovarian or prostate cancer can be studied using the corresponding sex, while other malignancies such as brain cancer, lung cancer, and cardiovascular disease should be explored by a mix of male/female animals that supports developing the most comprehensive clinical understanding for both sexes 156.

Animal conditioning and diet must be considered for preclinical theranostic experiments. In addition to adhering to all relevant animal protocols, including those set by Institutional Animal Care and Use Committees (IACUC), researchers should consider the influence of model-specific parameters on experimental outcomes. Furthermore, it is essential to consider the effects of any pretreatment, such as prior drug administration or the use of blocking agents. Whether animals are fasted or fed prior to radiopharmaceutical injection can have a significant impact on its uptake and biodistribution, especially if the radiotracer is designed based on certain metabolic pathways, such as [^18^F]FDG as a glycolysis biomarker 157,158. It should be considered if the nutrient balance in animal model diet might impact imaging or therapeutic experimental outcomes. For example, in the case of [^18^F]FDG, a fasting period of 5-6 hours in mice to achieve stable glucose levels is acceptable, and the readouts can be extrapolated to humans 159,160. In addition to fasting, circadian rhythm also has a role to play in modulating blood glucose levels, with a more pronounced effect of circadian rhythm on [^18^F]FDG uptake observed in the brain than in xenograft tumors 160. A normal or high fat diet may impact the biodistribution of some diagnostic radiopharmaceuticals, so this should be considered in experimental design depending on the type of targeting vector 161,162.

### 4.3. Preclinical imaging and pharmacokinetic analysis

After selecting a suitable animal model, preclinical SPECT or PET imaging and pharmacokinetic analysis can be performed to determine the compound biodistribution and potential suitability for TRT.

For any preclinical animal imaging protocol, there must be sufficient justification of scientific merit. Proper inclusion and exclusion criteria for the experiments should be defined so that all animals undergoing analysis are within normal parameters and controllable irregularities do not confound results. If a new imaging radionuclide is being used, the scanner should be calibrated with adequate energy detection windows, and phantom scans may be useful to validate imaging performance and determine appropriate imaging parameters prior to preclinical imaging 83,103.

SPECT or PET imaging should be performed at multiple time-points post-injection that reflect the expected residence time for the radiopharmaceutical, using dynamic or static scans 163. Dynamic imaging requires increased acquisition time and may be useful for the first hour after injection to understand the initial compound uptake dynamics, while static imaging can be used to assess biodistribution at extended timepoints. Dynamic scans are often used to quantify physiological processes such as organ perfusion or metabolism, and often use tracers that exhibit rapid uptake and clearance 144. Common applications of dynamic scans are cardiac imaging and neuroimaging 80. Static scans may be sufficient at extended time points for tracers with longer biological half-lives such as antibodies 164. These initial imaging procedures are important to determine the mode of radiopharmaceutical excretion, and any off-target binding that may occur in healthy tissue. Additionally, at set time points after radiopharmaceutical injection, animals can be sacrificed and dissected, with organs and tissues of interest harvested for radioactivity quantification to determine a precise biodistribution.

The method of radiopharmaceutical administration should be considered. Bolus injection is the traditional method of radiopharmaceutical administration, which provides an average uptake level throughout the scan duration. However, in applications such as neuroimaging, maintaining a steady plasma supply of radiopharmaceutical via continuous infusion injection may offer improved sensitivity to brain-state changes and improved temporal resolution 165.

A radiopharmaceutical can be administered to the body by tail vein intravenous injection, retro-orbital injection, subcutaneous, intraperitoneal, oral, or intranasal injection. The choice of administration methodology also plays a role in determining the radiopharmaceutical biodistribution and peak plasma activity time 166-168. For example, intraperitoneal, retro-orbital and tail vein injection methods can be used to administer [^18^F]FDG for imaging of normal physiological and tumor uptakes 167. Oral administration can be an alternative, however [^18^F]FDG administered orally distributes slower which leads to lower tumor uptake and a high gastrointestinal signal 167. For [^18^F]NaF imaging, both intra-peritoneal and intravenous injections are acceptable 166. The diseased target site should also be considered when choosing the route of administration. Administration of radiopharmaceuticals through intranasal injection gives an opportunity to target brain 168 whereas intraperitoneal injection route could be considered over intravenous injection route for targeting intraperitoneal tumors 169.

Regarding the administered radiopharmaceutical volume, different volume ranges are allowed for each injection route in rodents as highlighted in Table 2 170,171. For intranasal and retro-orbital injections, smaller volume is allowed which means higher molar/specific activity of the radiopharmaceutical should be used when intranasal or retro-orbital injections are being considered.

Absorption, distribution, metabolism, excretion (ADME) analysis can be performed to understand the complete pharmacokinetics of the administered radiopharmaceutical 172. Blood and excreta samples can be collected at set time points after injection and analyzed to determine the radioactivity present. This, coupled with mass spectrometric (LCMS), fluorometric, or spectrophotometric detection techniques can determine the distribution of radiopharmaceutical and its metabolites over time 172.

When administering theranostic radiopharmaceuticals and performing imaging procedures, animals are typically anesthetized to prevent pain and enable the procedure. It should be considered if anesthesia may impact the biodistribution of administered radiopharmaceuticals, particularly if the radiopharmaceuticals are involved in metabolism. Anesthetics like isoflurane, propofol, ketamine/xylazine or pentobarbital are commonly used in rodents and pigs. It was found that the cardiac uptake of [^18^F]FDG was significantly altered after exposure to ketamine/xylazine or pentobarbital or isoflurane as an anesthetic, suggesting that the choice of anesthetic can influence the observed radiopharmaceutical biodistribution 173. Further studies have shown that anesthesia produces significant effects on the brain and other organ systems 144. For example, isoflurane and propofol anesthesia resulted in decreased cortical [^18^F]FDG uptake in Sprague-Dawley rats 174. Since the duration of anesthesia can impact metabolism and therefore influence radiopharmaceutical uptake, the duration of anesthesia should be kept consistent throughout experiments. Similarly, body temperature can significantly affect radiotracer biodistribution, particularly for tracers of metabolic pathways.

Finally, blocking studies are valuable to evaluate specificity and can be used to address concerns regarding off target effects. Prior to imaging, there should be sufficient time to allow the blocking compound to accumulate at the target and reach equilibrium with the radiopharmaceutical 175.

### 4.4. Preclinical therapeutic analysis

After preclinical imaging and pharmacokinetic analysis have been performed with a favorable radiopharmaceutical biodistribution, the diagnostic radionuclide on the radiopharmaceutical can be swapped for a therapeutic radionuclide or a closely related radiotherapy molecule can be prepared. In initial therapeutic studies, animals with similar-sized tumors can be used to maintain more consistent results, since the state of tumor development along with its microenvironment can significantly impact the effects of TRT 176. The injected radioactivity per dose and the total number of doses should be considered with respect to the physical characteristics of the therapeutic radionuclide. Given similar therapeutic emissions, radionuclides with a shorter half-life (e.g. ^212^Pb or ^211^At) may require a greater injected dose than longer-lived radionuclides such as ^225^Ac. For different emissions, alpha-emitting radionuclides typically require significantly less injected activity compared to β^-^ emitters to achieve a similar therapeutic effect 177. A literature search should be performed to determine typical injected activities with similar compounds, and initial therapeutic experiments should use a smaller activity per dose, with dose increased gradually while monitoring for potential toxicity 178. A fractionated dose regimen may also be valuable to assess any difference in spreading out the total radioactivity over several injections, and if the fractionation reduces potential toxicity 179.

Depending on the model, it may be possible to pharmacologically upregulate the target prior to TRT. This has been performed previously 32, and may hold promise to enhance radionuclide delivery and efficacy provided that healthy tissues are not affected.

The tumor size at therapy initiation should be considered with respect to the radionuclide. While β^-^ emitters such as ^177^Lu or ^67^Cu possess longer electron emission pathlengths that can deposit dose in metastases (>2 mm in largest dimension), alpha particle or AE emitters possess shorter range emissions that may be more suitable for targeting circulating tumor cells or micrometastases (0.2-2 mm in largest dimension) 86. Therefore, after a therapeutic effect is verified, it may be informative to assess the radiopharmaceutical in groups of animals with several different tumor sizes.

The potential for combining targeted radionuclide therapy with immunotherapeutic agents such as immune checkpoint inhibitors has been explored, where combination therapy has improved TRT treatment durability and efficacy in multiple tumor models and motivated clinical trials 150. TRT can result in tumor cells undergoing immunogenic cell death, releasing tumor specific antigens in the process 148. This is accompanied by phenotypic changes in surviving cells with increased PD-L1, MHC-1, and Fas expression, and an alteration of innate immune cells in the tumor microenvironment. This can lead to an adaptive anti-tumor immune response, which can be enhanced by immune checkpoint inhibitors 150. If TRT/combination immune checkpoint therapies are investigated, control groups should ideally include both immunogenic and non-immunogenic mice with the same tumor model to help elucidate if any therapeutic enhancement may be due to the presence or lack of a functional immune system. Post therapy, if tumor clearance is observed, tumor rechallenge can be used to assess the durability of an immune response 180.

Diagnostic scans can be performed to evaluate initial response to therapy, and at extended timepoints to monitor treatment durability. The change in SUV value in molecular imaging scans after therapy may correspond with a reduction in disease and permits a systemic evaluation of treatment efficacy. In addition to using pre-therapy and post-therapy molecular imaging to assess therapeutic response, mice can be sacrificed at varying timepoints post-therapy to assess the distribution of therapeutic radiopharmaceutical. Organs can be harvested for gamma counting, and histologic analysis performed in dose-limiting organs to assess potential damage from the therapeutic doses 181. Blood and urine samples can also be taken throughout the therapeutic experiment, and for an extended duration post-therapy to assess changes in blood chemistry that may be indicative organ damage 182.

### 4.5. Dosimetry

One of the main selling points of theranostic radionuclide pairs is the promise of improved dosimetry estimation by using diagnostic imaging/ex-vivo biodistribution data and assuming identical biodistribution of the therapeutic counterpart 183. Using molecular imaging data and *ex vivo* radiopharmaceutical distribution data, dosimetry can be performed using a framework such as that developed by the Medical Internal Radiation Dose (MIRD) Committee 184. However, early versions of these frameworks were first established for diagnostic imaging purposes and are largely based on population-averaged data providing organ-level dosimetry. This was sufficient for the relatively low energy, long range radiation used in diagnostic radionuclides but is under increasing scrutiny for translation to therapeutics where precise dosimetry is a bottleneck challenge slowing translation. Within reason, errors in diagnostic dosimetry from oversimplification of complex biological systems could be overlooked because the dose was often overestimated and still maintained below a level where physiological damage could be expected 185. With therapeutic radionuclides, which consist of high-energy short-range particles selected for their ability to induce cytotoxic damage, this is no longer the case. The standard unit for reporting dose is the absorbed dose (D) measured in the SI unit Gray (Gy). The full formula for the absorbed dose varies in complexity depending on the level of precision but can be split into two broad terms: the time integrated activity (Ã) and the S-factor (S), as shown in Eq. 2.


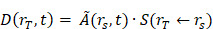

((Eq.2)

The S-factor gives the absorbed dose to a target region (r_T_) per decay of the radionuclide in the source region (r_s_), the time-integrated activity then sums up these contributions by giving the number of decays in the specified time (t). In the simplest static models, target-regions and sources cover entire organs, time can be integrated to infinity (until radionuclide decay), and physiological effects are modeled on population-average phantoms. With increasing complexity, target regions become voxel-sized (resolution of the PET scanner), and time-integration is done over time-activity curves for each region factoring in the pharmacokinetics of the radiopharmaceutical, all based on patient-specific scans 186-188.

Currently for preclinical evaluation, *ex vivo* based dosimetry is considered the “gold standard” due to its high sensitivity, reproducibility, simplicity, and the reduction of repeated anesthesia relative to preclinical imaging techniques 189. Activity data is converted to absorbed dose through tabulated calculations based on the radionuclide information (emission profile and half-life), and organ phantoms using tools such as the free MIRD_calc_ Excel-based platform 190 or commercially available OLINDA software 185. These calculations serve to evaluate the radioactive dose delivered to the target site, as well as surrounding healthy tissues and organs. The dosimetry data gathered with the diagnostic radionuclide can be extrapolated to a chemically similar/equivalent therapeutic radionuclide for an initial assessment of whether therapy might be viable. To obtain more nuanced dosimetry, autoradiography can evaluate the precise distribution of radioactivity within dissected tissues and organs, which can help inform dose-limiting activities and explain the root cause of potential toxicities 191. An evident draw-back of ex-vivo based biodistribution is that it provides only a snapshot in time of the radiopharmaceutical distribution, and extrapolations to obtain the absorbed dose (time-dependent) can quickly become inaccurate. Computational dosimetry methods 192 using multiple imaging time-points and time-activity curves that factor in the uptake and clearance profile of the radiopharmaceutical are an important area of development with a variety of commercial software tools available 193. Even with precise dosimetry and determination of radionuclide therapeutic efficacy in preclinical animal models, the task of translating this knowledge to human studies is non-trivial. In the absence of a more robust alternative, the first approach is to scale the activity concentration and expected therapeutic dose linearly based on the total-body mass, assuming (inaccurately) identical biology and general pharmacokinetics between the species. This crude model yields Eq. 3.


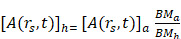

((Eq.3)

Where animal (a) and human (h) conversion of the activity concentration (A(r_s_,t)) occurs in the source region (r_s_) at some time-point (t), based on the body-mass (BM) ratio. It is the role of early-stage clinical trials to verify the therapeutic efficacy of this dosage mechanism 194, and this essential dosimetric analysis paves the way for clinical deployment.

## 5. Preparing for clinical translation

After validation in preclinical models, clinical translation can be considered based on an unmet clinical need that the theranostic radiopharmaceutical has potential to fill 195. Each targeting vector class has unique considerations for translation to clinical studies. For smaller vectors such as peptides, small changes in the amino acid sequence from existing compounds will require extensive analysis for regulatory approval. Unstable radiopharmaceuticals typically do not proceed to clinical trials unless their degradation is part of a specific metabolic pathway 196. Larger vectors such as antibodies may be less affected by functionalization (e.g. chelator attachment) and may not require as extensive analysis provided that pharmacokinetic data exists for the unmodified antibody and fully characterized GMP-grade precursor is available. For more novel targeting vectors such as liposomes or nanoparticles, there may be little regulatory guidance. Prior to human studies, as much data on the expected behavior of the compound in humans should be gathered as possible 196.

The radiopharmaceutical synthesis should be performed according to current good manufacturing practices (cGMP), with chemistry manufacturing and controls (CMC) in place 197. These cGMP requirements are crucial to maintain the quality and safety of radiopharmaceuticals throughout the entire production process and ensure that the required product specifications (e.g. appearance, pH, endotoxins, radiochemical identity and purity, radionuclidic identity, chemical purity, residual solvents, sterility) are reproducible and consistent among batches. General guidelines and resources on cGMP for radiopharmaceuticals have been published by organizations such as the International Atomic Energy Agency (IAEA) and the World Health Organization (WHO), however local regulations must be adhered to. CMC should ensure that radiopharmaceutical synthesis occurs in a safe manner with a consistent quality. The CMC documents should be clearly written in a concise manner that allows personnel to synthesize the precursor and radiopharmaceutical without any difficulties. All abbreviations in the CMC should be clearly defined (including “industry standard” abbreviations), schematic diagrams and pictures should be included whenever there may be the possibility for misinterpretation, and links to useful references should be provided to enhance clarity and provide additional background information.

Product characterization and testing should determine the stability and key radiochemical parameters (e.g. pH, radionuclidic purity, HPLC/TLC analysis) following a successful radiopharmaceutical synthesis 133. The required chemical inventory for manufacturing will need to be prepared and maintained, specification sheets and comprehensive certificates of analysis (CofA) that include preparation/expiry dates and other radiochemical parameters that the precursor and subsequent radiopharmaceutical must meet. For all new compounds, toxicity data has to be generated by conducting an anticipated range of human equivalent doses in rodents and should be analyzed for any toxicity over time by monitoring animal behavior, weight, vitals, various blood biomarkers, liver, and kidney function tests to ensure the safety of the new drug before performing human studies 198.

Once the clinical manufacturing processes have been validated, there are additional aspects to consider (these will vary depending on the jurisdiction) 199. For example, in the USA, an Institutional Review Board (IRB) submission needs to be made and approved prior to participant recruitment, and an Investigational New Drug (IND) application must be filed with the Food and Drug Administration (FDA) 200. Additional aspects to consider include patient recruitment, media advertisement, informed consent, scripts for study coordinators and receptionists, handling of sensitive information, study timeline, and project management. Any team supporting a clinical trial should have multi-disciplinary personnel including a chemistry/radiochemistry expert, an automation and cGMP expert, a biology expert, a clinical expert, excellent support staff (e.g. technical study coordinator), staff to assist with compliance (cGMP) and approvals (IRB, FDA), and dedicated project management.

## 6. Outlook and conclusions

In summary, there is significant interest and growth in theranostic radiopharmaceutical development and clinical application due to their ability to precisely diagnose and treat systemic malignancies that are refractory to standard-of-care treatments. Many oncologic targets can benefit from theranostic applications, and other theranostic targeting vectors such as microbial-targeting radiopharmaceuticals may also hold promise. Supply constraints associated with therapeutic radionuclides, particularly alpha emitters (e.g. ^225^Ac, ^212^Pb, ^211^At) should ease as additional production facilities come online and existing facilities enhance their output. While existing vectors for targets such as SSTR2 on neuroendocrine tumors and PSMA on prostate cancer have shown significant promise in theranostic clinical trials, they can still be improved. Next-generation SSTR2 and PSMA theranostic targeting vectors hold potential to further improve antitumor efficacy through improved tumor uptake and reduced uptake in off-target tissues. More recent vectors, such as fibroblast activation protein inhibitors, have potential to offer theranostics for a multitude of cancers. Additionally, the combination of immune checkpoint therapy with radiopharmaceutical theranostics has shown synergistic effects in preclinical studies, with multiple clinical trials planned for immune checkpoint inhibitors in combination with radiopharmaceutical theranostics. Overall, the theranostic approach can significantly improve patient quality of life and survival and overcome resistance in systemic diseases to existing standard of care treatments.

## Author Contributions

B.J.B.N. prepared the manuscript draft; V.K., A.B., B.J.B.N., J.D.A., F.W., and M.K.P. performed review and editing. All authors have read and agreed to the published version of the manuscript.

## Figures and Tables

**Table 1:** Selection of transgenic animal models explored for several common disease targets.

Imaging Target	Transgenic Model	Reference
Neurodegenerative disease (β-Amyloid and Tau Imaging)	APPswe, APP/PS1, 3 × Tg, 5 × FAD, Tg2576, and APP23, rTG4510, PS19, P301S	152
Prostate Cancer	TRAMP, ARR_2_PB-c-Myc, PTEN^+/-^, PTEN^+/-^/Nkx3.1^-/-^, PB-Cre4/Trp53^L2/L2^/ /Rb^L2/L2^, PB-Cre4/PTEN^L2/L2^/ SMAD4^L2/L2^, PB-Cre4/PTEN^L2/L2^/ Trp53^L2/L2^/SMAD4^L2/L2^ and PSA-Cre-ER^T2^/PTEN^L2/L2^	153
Breast Cancer	BRCA1, c-MET, c-MYC, CDC37, ERBB2/HER2/neu, HRAS, NOTCH4, PIK3CA, PTEN, PYMT, RB1, SV40, TP53, WNT1, TTA, TGFβR2, IGF-IR transgenic mice	154
Melanoma	SV40 transgenic, HRAS, NRAS, KRAS, P53, LKB1 P16INK4A mutants	155

**Table 2:** Permissible injection volume in rodents for different administration routes 170,171.

Route of Administration	Permissible volume
Tail vein	Up to 5 mL/kg
Retro-orbital	50 µL in rodents
Subcutaneous	Up to 5 mL/kg per site
Intraperitoneal	Up to 10 mL/kg
Oral	10 ml/kg for mice and 10-20 ml/kg for rats
Intranasal	35-50 µL in rodents

## Abbreviations

ADME: absorption, distribution, metabolism, excretion
AE: Auger electron
ASO: antisense oligonucleotides
BCA: bicinchoninic acid
BDG: beta-(1,3)-glucan
CADD: computer-aided drug design
CAR-T: chimeric antigen receptor T-cell therapy
CCK2R: cholecystokinin-2 receptor
CD4bs: CD-4 binding site
cGMP: current good manufacturing practices
CMC: chemistry manufacturing and controls
CofA: certificate of analysis
CXCR4/CXCL12: chemokine receptor type 4 and 12
DSB: double-strand break
EPR: enhanced permeability and retention
Fab: antigen binding fragments
FAB4: fatty acid-binding protein 4
FAP: Fibroblast activation protein
FASN: fatty acid synthase
Fcabs: Fc fragments with antigen binding
FDG: fluorodeoxyglucose
FRβ: folate receptor beta
GlcCer: glucosylceramide
GLP-1: glucagon-like peptide 1
GMP: good manufacturing practices
GnRH-R: gonadotropin releasing hormone receptor
GP120: glycoprotein 120
GP41: glycoprotein 41
GRPR: gastrin-releasing peptide receptor
GXM: glucuronoxylomannan
HER2: human epidermal growth factor receptor 2
HIV: human immunodeficiency virus
HNSCC: head and squamous cell carcinoma
HPLC: high performance liquid chromatography
HSP60: heat shock protein 60
IAEA: International Atomic Energy Agency
IC_50_: half-maximal inhibitory concentration
ICP-MS: inductively-coupled plasma mass spectroscopy
ICP-OES: inductively-coupled plasma optical emission spectroscopy
IND: investigational new drug
LET: linear energy transfer
LF: lethal factor
MC1R: melanocortin receptor subtype 1
MHC-1: major histocompatibility complex
MIRD: Medical Internal Radiation Dose
MPS: mononuclear phagocytic system
NK1R: neurokinin-1 receptor
NOTA: 2,2′,2”-(1,4,7-triazacyclononane-1,4,7-triyl)triacetic acid
NTR1: neurotensin receptor 1
PA: protective antigen
PD-1: programmed cell death protein 1
PD-L1: programmed death-ligand 1
PET: positron emission tomography
PPS8: pneumococcal capsular polysaccharide 8
PSMA: prostate specific membrane antigen
SB: specification book
SPE: solid phase extraction
SPECT: single photon emission computed tomography
SSB: single-strand break
SSTR2: somatostatin receptor 2
STEAP1: six transmembrane epithelial antigens of the prostate 1
SUV: standardized uptake value
TCMC: 2-[4,7,10-tris(2-amino-2-oxoethyl)-1,4,7,10-tetrazacyclododec-1-yl]acetamide
TLC: thin layer chromatography
TRT: Targeted radionuclide therapy
TSPO: translocator protein
VEGF: vascular endothelial growth factor
VPAC1: vasoactive intestinal peptide receptor 1
WHO: World Health Organization
WTA: wall teichoic acid
